# Usefulness of the Texture Signatures Based on Multiparametric MRI in Predicting Growth Hormone Pituitary Adenoma Subtypes

**DOI:** 10.3389/fonc.2021.640375

**Published:** 2021-07-07

**Authors:** Chen-Xi Liu, Li-Jun Heng, Yu Han, Sheng-Zhong Wang, Lin-Feng Yan, Ying Yu, Jia-Liang Ren, Wen Wang, Yu-Chuan Hu, Guang-Bin Cui

**Affiliations:** ^1^ Department of Radiology, Tangdu Hospital, Air Force Medical University (Fourth Military Medical University), Xi’an, China; ^2^ Functional and Molecular Imaging Key Lab of Shaanxi Province, Xi’an, China; ^3^ Department of Neurosurgery, Tangdu Hospital, Air Force Medical University (Fourth Military Medical University), Xi’an, China; ^4^ Faculty of Medical Technology, Shaanxi University of Traditional Chinese Medicine, Xianyang, China; ^5^ GE Healthcare China, Beijing, China

**Keywords:** growth hormone pituitary adenoma, pathological subtype, texture analysis, magnetic resonance imaging (MRI), densely granulated somatotroph adenoma, sparsely granulated somatotroph adenoma

## Abstract

**Objective:**

To explore the usefulness of texture signatures based on multiparametric magnetic resonance imaging (MRI) in predicting the subtypes of growth hormone (GH) pituitary adenoma (PA).

**Methods:**

Forty-nine patients with GH-secreting PA confirmed by the pathological analysis were included in this retrospective study. Texture parameters based on T1-, T2-, and contrast-enhanced T1-weighted images (T1C) were extracted and compared for differences between densely granulated (DG) and sparsely granulated (SG) somatotroph adenoma by using two segmentation methods [region of interest 1 (ROI_1_), excluding the cystic/necrotic portion, and ROI_2_, containing the whole tumor]. Receiver operating characteristic (ROC) curve analysis was performed to determine the differentiating efficacy.

**Results:**

Among 49 included patients, 24 were DG and 25 were SG adenomas. Nine optimal texture features with significant differences between two groups were obtained from ROI_1_. Based on the ROC analyses, T1WI signatures from ROI_1_ achieved the highest diagnostic efficacy with an AUC of 0.918, the accuracy, sensitivity, specificity, positive predictive value (PPV), and negative predictive value (NPV) were 85.7, 72.0, 100.0, 100.0, and 77.4%, respectively, for differentiating DG from SG. Comparing with the T1WI signature, the T1C signature obtained relatively high efficacy with an AUC of 0.893. When combining the texture features of T1WI and T1C, the radiomics signature also had a good performance in differentiating the two groups with an AUC of 0.908. In addition, the performance got in all the signatures from ROI_2_ was lower than those in the corresponding signature from ROI_1._

**Conclusion:**

Texture signatures based on MR images may be useful biomarkers to differentiate subtypes of GH-secreting PA patients.

## Introduction

Pituitary adenomas (PAs) represent approximately 14% of primary intracranial and central nervous system tumors (1, 2). Most PAs have a high secretory function and lead to a series of clinical symptoms (3). The incidence rate of growth hormone (GH)-secreting PAs is next to the non-functioning PAs and prolactin adenomas (4), which is the majority cause of acromegaly (5). GH-secreting PAs are divided into densely granulated (DG) and sparsely granulated (SG) adenoma pathologically based on granulation pattern (6). At present, surgery is the first-line treatment for GH adenomas (7–9). Compared with DG somatotroph adenomas, SG adenomas own a larger size, stronger invasion, and poorer prognosis (10). The above characteristics determine the differences in the scope or approach of tumor resection and even affect the outcome of transsphenoidal surgery (8, 9, 11). Therefore, it is essential to predict the pathological subtypes before formulating optimum treatment regimens accurately.

At present, the diagnosis and evaluation of PAs mainly depend on magnetic resonance imaging (MRI). Several studies have revealed that MR signal could predict some histological features of PAs (12–15). DG adenomas are often manifest as hypointense on T2-weighted images (T2WI) (12), and there is a significant correlation between T2WI signal intensity and the pathological subtypes of GH adenomas (16). Besides, T2 high signal GH adenoma is a higher invasion and larger size than T2WI low signal GH adenoma (1–3, 15, 17). However, these studies are based on single T2WI signal features, which cannot comprehensively reflect the morphological characteristics in identifying PA subtypes.

Texture analysis reveals the distribution of signal intensity at a pixel level within a tumor to quantify the tumor heterogeneity (18). Compared with morphological features of conventional imaging, texture parameters provide more quantitative information based on the whole tumor and reflect the biological behavior of the tumor. A series of texture analysis studies have demonstrated high performance in the differential diagnosis (19), evaluation of soft consistency (20) and aggressiveness (21) in pituitary macroadenoma, identification of granulation pattern in GH adenoma (22), and predicting treatment response in patients with PA (23, 24). However, it remains mostly unknown whether texture analysis based on multi-sequence MRI can improve the efficacy in predicting the GH-secreting PA subtypes.

In the present study, we aimed to evaluate the potential values of MRI texture parameters in predicting the pathological subtypes of GH-secreting PAs preoperatively.

## Materials and Methods

### Patients

This study was approved by the medical ethics committee of Tangdu Hospital of the Fourth Military Medical University (NO. TDLL-KY202011-04), and informed consent was waived according to its retrospective nature. This study was conducted in accordance with the Declaration of Helsinki.

**Figure 1:**
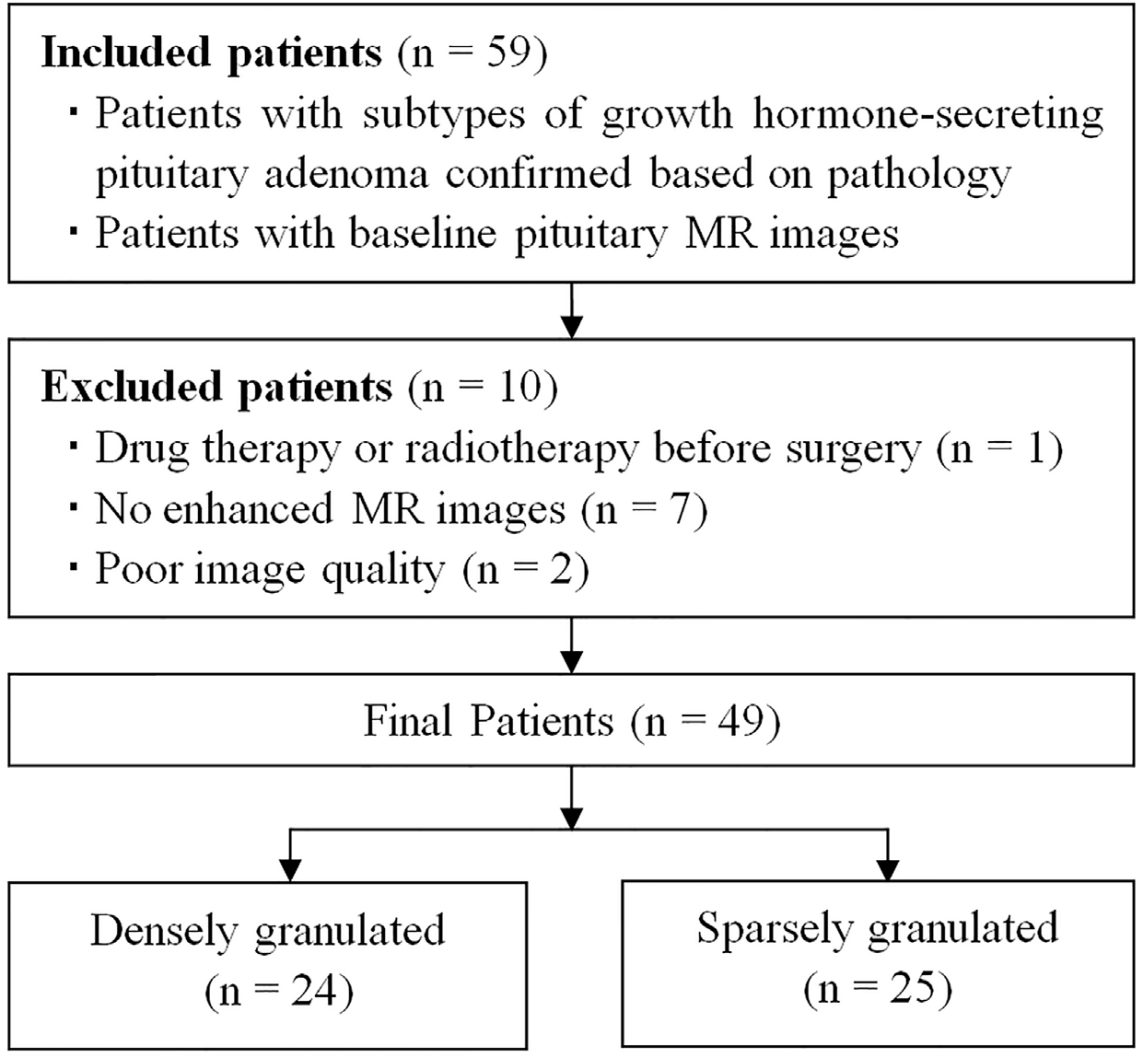
Flow diagram of patient selection and grouping.

Between October 2015 and July 2020, a total of 54 patients with acromegaly underwent pituitary MRI were initially included based on the following inclusion criteria: (1) patients with GH-secreting PA proved by pathological analysis, and the subtypes confirmed by immunohistochemical staining; (2) patients with baseline pituitary MR images. The exclusion criteria were as follows: (1) patients with drug therapy or radiotherapy before surgery (n = 1); (2) no enhanced MR images (n = 7); (3) poor image quality or obvious artifact (n = 2). The final study population was comprised of 49 patients (23 men, 26 women) with GH-secreting PAs, and the patients were divided into DG (n = 24) and SG (n = 25) groups (**Figure 1** and **Table 1**). The serum levels of GH and insulin-like growth factor-1 were detected in all patients at baseline.

**Table 1:** Demographic characteristics of 49 patients with growth hormone pituitary adenoma.

	DG (n = 24)	SG (n = 25)	P
Gender (n, M/F)	14/10	9/16	0.117
Age (years)	42.13 ± 11.71	39.96 ± 12.75	0.539
GH (μg/L)	61.34 ± 77.41	39.10 ± 35.10	0.198
IGF-1(nmol/L)	471.00 ± 138.93	441.43 ± 134.38	0.462
Tumor volume (cm^3^)	9.86 ± 13.81	15.91 ± 14.11	0.136

Values denote as (mean ± SD) unless otherwise indicated. DG, densely granulated; SG, sparsely granulated; GH, growth hormone; IGF-1, insulin-like growth factor-1.

### MR Image Acquisition

All preoperative MRI examinations were performed using a 3.0-T whole-body system (MR750, GE Healthcare, Milwaukee, WI, USA) with a 40-mT/m maximum gradient capability and a standard receive-only head coil. Pituitary MRI protocols included precontrast (T1WI: repetition time [TR]/echo time [TE], 441 ms/Minimum; field of view [FOV], 220 mm; matrix size, 288 × 224; number of excitation [NEX], 4; slice thickness, 1.6 mm; gap, 0.3 mm), coronal T2-weighted imaging (T2WI: TR/TE, 441 ms/Minimum; FOV, 220 mm; matrix size, 288 × 224; NEX, 4; slice thickness, 1.6 mm; gap, 0.3 mm), sagittal T2WI (TR/TE, 441 ms/Minimum; FOV, 220 mm; matrix size, 288 × 224; NEX, 4; slice thickness, 1.6 mm; gap, 0.3 mm). Subsequently, coronal and sagittal contrast-enhanced T1WI (T1C: imaging parameters were the same with precontrast sequences) were obtained 3 min 16 s after intravenous bolus injection of 0.1 mmol/kg bodyweight of gadodiamide (Omniscan; GE Healthcare, Co. Cork, Ireland) at a rate of 0.2 ml/s.

### Tumor Segmentation

**Figure 2:**
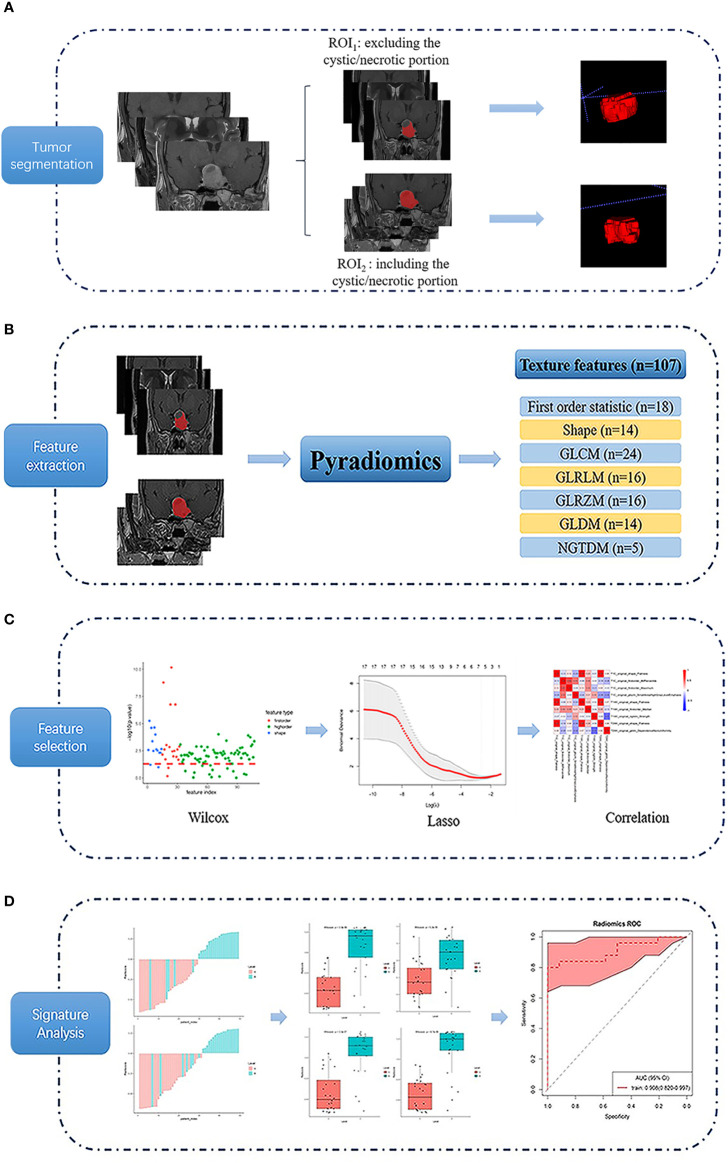
Flow chart of the texture analysis process. Firstly, a region of interest (ROI) was drawn using 2 segmented methods (ROI_1_, excluding the cystic/necrotic portion, and ROI_2_, containing the whole tumor area) on coronal T1-weighted, T2-weighted, and contrast-enhanced T1-weighted images **(A)**. Then 107 texture features were extracted **(B)**, and the least absolute contraction and selection operator (LASSO) was used for feature selection **(C)**. Finally, the differentiating performance of the optimal features was analyzed **(D)**.

All image data were stored in DICOM format after the anonymization process. Then the raw data were imported into the ITK-SNAP software (version 3.6.0 www.itksnap.org). An experienced radiologist reviewed all the non-contrast and contrast-enhanced MR images carefully to determine the margin of adenoma. A region of interest (ROI) was drawn manually around the entire cross-sectional tumoral region in all consecutive slices of the tumor on coronal T1C images in a slice-by-slice way according to two segmented methods (ROI_1_, excluding the cystic/necrotic portion, appeared as no enhanced area on T1C images, and ROI_2_, containing the whole tumor area), and maintain the ROI edge 1–2 mm away from the tumor margin for minimizing the partial volume effect. The ROI of coronal non-contrast T1WI and T2WI were obtained by matching the ROI of coronal T1C images (**Figure 2A**). The mean ROI size of the lesions was 4,644.6 ± 4,726.4 mm³ for ROI_1_, and 5,067.9 ± 4,790.0 mm³ for ROI_2_, with the same range of 329.3–17,360.0 mm³.

### Feature Extraction and Analysis

Features were extracted from the Coronal images of T1, T2WI, and T1C, respectively. The PyRadiomics platform (25) is used to extract 107 quantitative texture features (**Figure 2B**), which can be divided into seven categories: (1) Shape (n = 14), (2) First Order Statistics (n = 18), (3) Gray Level Cooccurrence Matrix (n = 24), (4) Gray Level Run Length Matrix (n = 16), (5) Gray Level Size Zone Matrix (n = 16), (6) Neighboring Gray Tone Difference Matrix (n = 5), and (7) Gray Level Dependence Matrix (n = 14). Details of the quantitative features extracted in this study are presented in the **Supplemental File** ***(Texture Parameters.PDF)***.

The least absolute contraction and selection operator (LASSO) logistic regression algorithm was used to identify the most significant predictive features from 107 features. A radiomics signature was constructed using the radiomics score, which was calculated as a linear combination of selected features that were weighted by their respective LASSO coefficients. To provide a robust generalized performance of a model that best fits the observed data, tenfold cross-validation was performed (**Figure 2C**).

### Statistical Analysis

Statistical analyses of patient demographic data were performed with IBM SPSS 20.0 software (IBM Corp, Chicago, IL, USA). Differences in patients’ demographic characteristics between DG and SG were conducted with the Pearson chi-square test or independent-samples T-test. Texture feature analyses were implemented using R statistical software (version 3.4.2). LASSO logistic regression was performed using the “glmnet” package. Wilcoxon test was done with the “base” package. The receiver operating characteristic (ROC) curves were plotted using the “pROC” package. Using pathologically proven PA subtypes as the gold standard, the area under the curve (AUC), accuracy, sensitivity, specificity, positive predictive value (PPV), and negative predictive value (NPV) of texture signatures were calculated in differentiating the DG and SG. Decision curve analysis (DCA) was performed with the function “dca.R” (**Figure 2D**). P < 0.05 was considered as statistical significance.

## Results

### Demographic Characteristics

**Table 1** exhibited the baseline characteristics of 49 GH-secreting PA patients. Final patients were consisted of 24 DG (male, 14; female, 10; mean age, 42.13 ± 11.71 years) and 25 SG (male, 9; female, 16; mean age, 39.96 ± 12.75 years). There were no significant differences in gender, age, tumor volume, serum levels of GH, and insulin-like growth factor-1 between DG and SG (P > 0.05).

### Comparison of Texture Parameters Between Groups DG and SG

After feature selection, nine optimal texture features with significant differences between two groups were obtained from ROI_1_, including *T1C_Flatness*, *T1C_90Percentile*, *T1C_Maximum*, *T1C_SmallAreaHighGrayLevelEmphasis*, *T1WI_Flatness*, *T1WI_Median*, *T1WI_Strength*, *T2WI_Flatness*, and *T2WI_DependenceNonUniformity*. Four optimal features were obtained from ROI_2_, including *T1WI_Flatness, T1WI_InterquartileRange*, *T1WI_ RootMeanSquared*, and *T1WI_ Lowgray level zone emphasis.*

**Figure 3:**
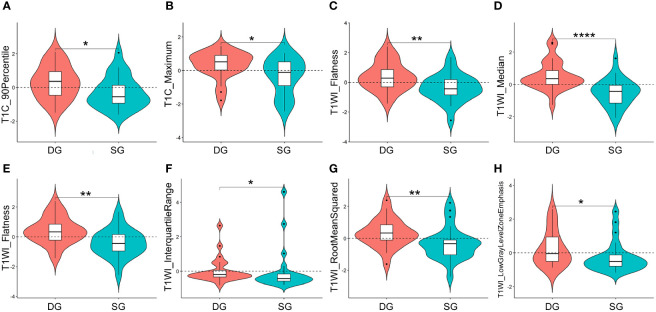
Violin plots for comparisons of representative texture parameters between densely granulated (DG) and sparsely granulated (SG) growth hormone-secreting adenomas, which extracted from ROI_1_: *TIC_90Percentile* **(A)**, *T1C_Maximum* **(B)**, *T1WI_Flatness* **(C)**, and *T1WI_Median* **(D)**, and ROI_2_: *T1WI_Flatness* **(E)**, *T1WI_InterquartileRange* **(F)**, *T1WI_ RootMeanSquared* **(G)**, and *T1WI_ Lowgray level zone emphasis* **(H)**. *P < 0.05, **P < 0.01, ****P < 0.001. ROI, region of interest; ROI_1_, excluding the cystic/necrotic portion; ROI_2_, containing the whole tumor; T1C, contrast-enhanced T1WI.

As showed in **Figure 3**, the representative texture features extracted from ROI_1_ and ROI_2_ were compared between DG and SG. The optimal features from ROI_1_: significant difference was found in *TIC_90Percentile* (P < 0.05), *T1C_Maximum* (P < 0.05), *T1WI_Flatness* (P < 0.01), and *T1WI_Median* (P < 0.001) between two groups. As for the optimal texture features from ROI_2,_ there were significant differences in *T1WI_Flatness* (P < 0.01), *T1WI_InterquartileRange* (P < 0.05), *T1WI_ RootMeanSquared* (P < 0.01), and *T1WI_ Lowgray level zone emphasis* (P < 0.05) between two groups.

### Efficacy Analysis of Texture Signatures in Differentiating DG From SG

**Figure 4:**
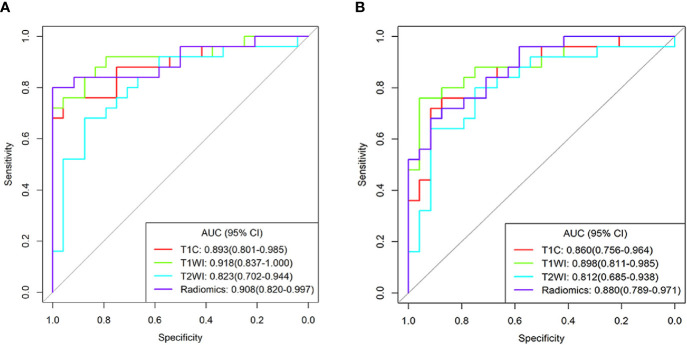
Receiver operating characteristic curves for the differentiating performance of texture signatures from T1C, T1WI, T2WI, and Radiomics between densely granulated and sparsely granulated growth hormone-secreting adenomas. Texture features extracted from region of interest1(ROI_1_) **(A)** and ROI_2_ **(B)**, respectively. Radiomics signatures based on the radiomics score, combined the texture parameters from T1C and T1WI. ROI_1_, excluding the cystic/necrotic portion; ROI_2_, containing the whole tumor; AUC, area under curve; CI, confidence interval; T1C, contrast-enhanced T1WI.

Based on the ROC analyses, T1WI signatures from ROI_1_ achieved the highest diagnostic efficacy with an AUC of 0.918; the accuracy, sensitivity, specificity, PPV, and NPV were 85.7, 72.0, 100.0, 100.0, and 77.4%, respectively, for distinguishing DG from SG. Comparing with the signature from T1WI, the T1C signature also obtained relatively high efficacy with an AUC of 0.893. When combining the texture features of T1WI and T1C, the radiomics signatures had a good performance in differentiating the two groups with an AUC of 0.908. However, the efficacies obtained in all the signatures from ROI_2_ were lower than those in the corresponding signature from ROI_1_ (**Table 2** and **Figure 4**).

**Table 2:** Performance of texture signatures in differentiating the growth hormone pituitary adenoma subtypes.

Cohorts	Signatures	AUC	Accuracy	Sensitivity	Specificity	PPV	NPV
ROI_1_	T1C	0.893 (0.801–0.985)	0.857 (0.728–0.941)	0.760 (0.520–0.881)	0.958 (0.583–1.000)	0.950 (0.929–0.957)	0.793 (0.700–0.800)
	T1WI	0.918 (0.837–1.000)	0.857 (0.728–0.941)	0.720 (0.560–0.920)	1.000 (0.792–1.000)	1.000 (1.000–1.000)	0.774 (0.731–0.774)
	T2WI	0.823 (0.702–0.944)	0.776 (0.634–0.882)	0.680 (0.320–0.880)	0.875 (0.583–1.000)	0.850 (0.727–0.880)	0.724 (0.636–0.750)
	Radiomics	0.908 (0.820–0.997)	0.898 (0.778–0.966)	0.800 (0.640–0.960)	1.000 (0.375–1.000)	1.000 (1.000–1.000)	0.828 (0.643–0.828)
ROI_2_	T1C	0.860 (0.756–0.964)	0.816 (0.680–0.912)	0.720 (0.240–0.880)	0.917 (0.582–1.000)	0.900 (0.750–0.917)	0.759 (0.666–0.774)
	T1WI	0.898 (0.811–0.985)	0.857 (0.728–0.941)	0.760 (0.320–0.921)	0.958 (0.667–1.000)	0.950 (0.889–0.958)	0.793 (0.727–0.800)
	T2WI	0.812 (0.685–0.938)	0.776 (0.634–0.882)	0.640 (0.120–0.841)	0.917 (0.583–1.000)	0.889 (0.600–0.913)	0.710 (0.609–0.727)
	Radiomics	0.880 (0.789–0.971)	0.796 (0.657–0.898)	0.680 (0.400–0.880)	0.917 (0.583–1.000)	0.895 (0.833–0.917)	0.733 (0.636–0.750)

The value is expressed as a percentage (%) and 95% confidence interval. ROI_1_, region of interest excluding the cystic/necrotic portion appeared as no enhanced area on T1C images; ROI_2_, region of interest containing the whole tumor area; AUC, area under curve; PPV, positive predictive value; NPV, negative predictive value; T1C, contrast-enhanced T1WI.

### Decision Curve Analysis

**Figure 5:**
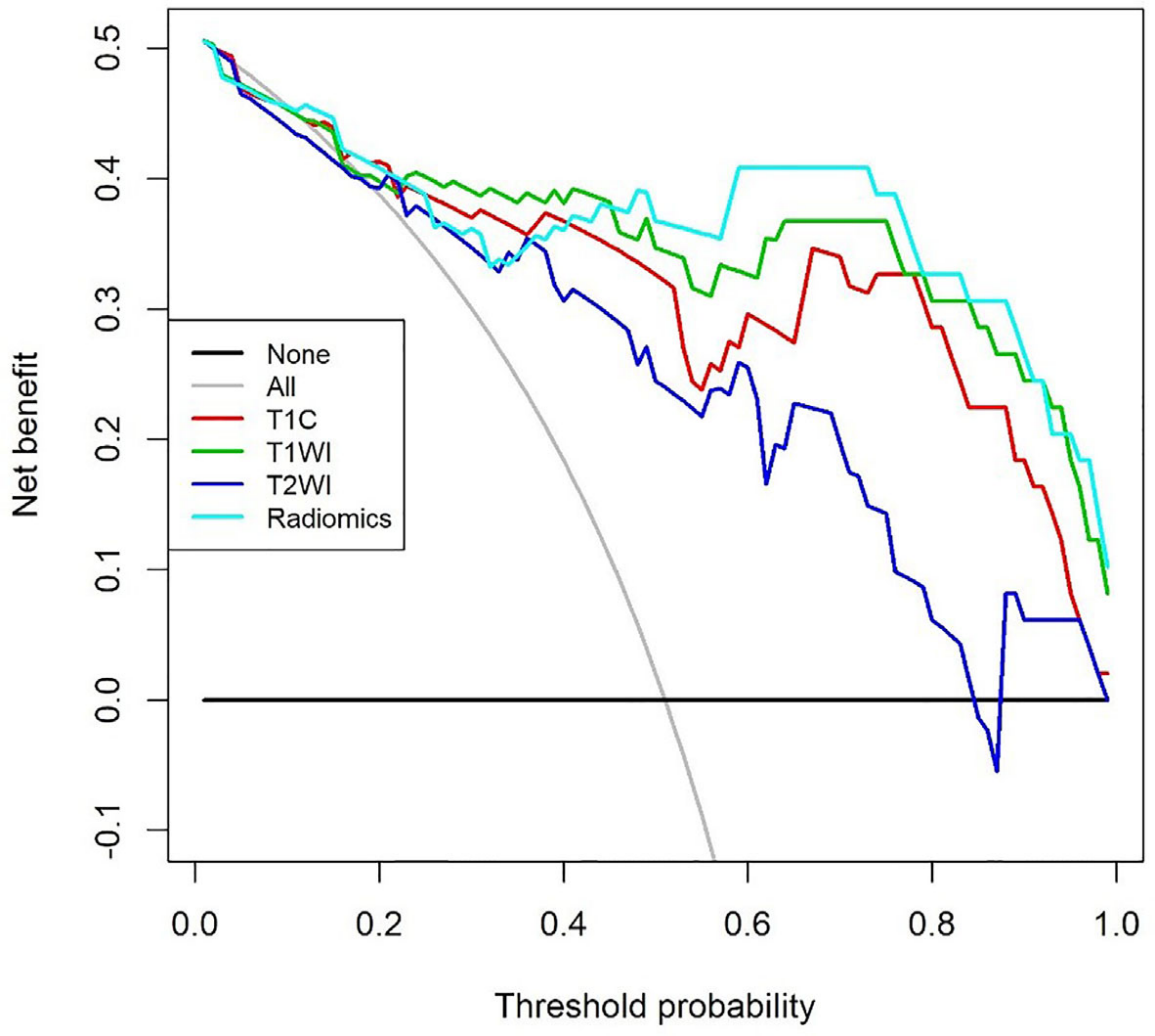
Decision curve analysis for models from contrast enhanced T1WI (T1C), T1WI, T2WI, and Radiomics signatures in differentiating growth hormone-secreting adenoma subtypes. The decision curve showed that the prediction model had a better net benefit than either the treatment or no treatment schemes when the threshold probability was 0.254 to 0.798.

The DCA for models from T1C, T1WI, T2WI, and radiomics signatures in differentiating GH-secreting adenoma subtypes was presented in **Figure 5**. The DCA showed that our prediction model had a better net benefit than either the treatment or no treatment schemes when the threshold probability was 0.254 to 0.798.

## Discussion

In this study, we evaluated the significance of MRI based texture signatures in differentiating the GH-secreting PA subtypes. As a result, significant differences in optimal texture features were demonstrated between groups DG and SG, and good performance was obtained in differentiating two subtypes by using texture signatures from T1W, T1C, and T2W images. Therefore, the MRI based texture signatures can be used for predicting pathological subtypes of GH-secreting PA preoperatively.

The relevant literature demonstrated that DG adenomas are mostly hypointense on T2W images and correlate with a smaller adenoma size, higher GH, and IGF-1 levels (14, 26, 27). In this study, the smaller size, higher GH and IGF-1 were found in DG adenomas than those of SG adenomas, although no significant statistical difference existed. The grade of parasellar extension was directly related to the tumor size (28). Therefore, SG type adenomas tend to be larger and more aggressive than DG type adenomas.

A series of studies revealed that DG somatotroph adenomas often show hypo- or iso-intensity on T2WI in the visual qualitative evaluation of adenomas, which helps differentiate DG from SG (17, 29). Comparing with morphological analysis of MRI, texture parameters provide more detailed and quantitative information of the whole tumor (18, 30). In this study, we got nine optimal texture features after feature selection. The *Flatness* value of DG was significantly higher than that of SG in all MR sequences. *Flatness* reflects the smoothness of the tumor surface, indicating that the tumor margin of DG is smoother, which is consistent with the biological characteristics of lower malignancy in DG. Previous studies mainly focused on the correlation between T2 signal intensity and tumor granulation pattern in patients with GH adenomas and confirmed the potential value of tumor signal intensity in distinguishing DG from SG (31, 32). Interestingly, there were significant differences between DG and SG in *Maximum*, *Median*, and *90Percentile* features in our study, which belong to a category of the first-order feature and describe the distribution of voxel intensities within the image region. Therefore, the above results may reflect the difference of tumor spatial heterogeneity between the two subtypes, which may be related to the differences in MR signal characteristics between the two subtypes.

A recent study revealed that radiomic features based on T2WI were helpful in predicting the granulation pattern of GH-secreting adenoma patients, with better performance than qualitative assessment or relative signal intensity (rSI) evaluation (AUC: 0.834 *vs.* 0.647) (22). Similarly, our results also showed good performance on T2WI signatures to distinguish the adenoma subtypes, with an AUC of 0.823. However, unlike previous studies, we also analyzed the efficacy of T1WI and T1C signatures in identifying PA subtypes. The results showed that the AUC both for T1WI and T1C signature was higher than that of T2WI (AUC: 0.918 and 0.893 *vs.* 0.823). In addition, radiomics signatures combining T1WI and T1C also achieved high performance with an AUC of 0.908 in differentiating the DG from SG. Interestingly, texture parameters combining two MR sequences did not improve the differentiating efficacy, and the reason for such a result could be that T1WI and T1C are from the same MR sequence. Furthermore, according to the decision curve, the radiomics model is most beneficial to predict GH-secreting PA subtypes.

Besides, in our study, we extracted texture features manually using two tumor segmentation methods (ROI_1_, excluding the cystic/necrotic portion that appeared as no enhanced area on T1C images, and ROI_2_, containing the whole tumor area). The results indicated that the efficacies obtained in all the signatures from ROI_1_ were higher than those in corresponding signatures from ROI_2_. Compared with the manual and time-consuming slice-by-slice segmentation process, automatic or semi-automatic tools are an alternative, which can be significantly faster and less user intensive (33). However, this study compared the efficacy of the two segmentation methods for ROI_1_ and ROI_2_, and obviously invasion of cavernous sinus was found in a number of cases. For the accuracy of segmentation, we adopted the manual segmentation method in this study.

## Limitations

There are several limitations of this study. First, the sample size was relatively small. Second, the study did not perform external verification, so the repeatability of the predicted signatures was not confirmed. Finally, as a preliminary study to explore the possibility of using MR texture analysis for obtaining quantitative biomarkers of GH-secreting adenoma, and further radiomics study with a larger sample and external validation are needed to clarify this issue.

## Conclusion

In conclusion, as a useful and non-invasive biomarker, texture signatures derived from MRI texture analysis could help discriminate pathological subtypes of GH-secreting adenomas before surgery, potentially used in clinical practice for individualized treatment strategies.

## Data Availability Statement

The raw data are not publicly available due to them containing information that could compromise research participant privacy/consent.

## Ethics Statement

The studies involving human participants were reviewed and approved by the Medical Ethics Committee of Tangdu Hospital of the Fourth Military Medical University. Written informed consent for participation was not required for this study in accordance with the national legislation and the institutional requirements.

## Author Contributions

G-BC and Y-CH conceived the study. C-XL, YH, and S-ZW participated in the study design. C-XL, YH, S-ZW, L-JH, L-FY, YY, J-LR, WW, and Y-CH performed the data acquisition. C-XL and YH participated in the statistical analyses. All authors participated in the data interpretation. C-XL drafted the first version of the report. All authors contributed to the article and approved the submitted version.

## Funding

This work was supported by the Key research and development plan of Shaanxi province (No. 2019ZDLSF02-07) and the Science and Technology Innovation Development Foundation of Tangdu Hospital (No. 2017LCYJ004).

## Conflict of Interest

Author J-LR was employed by company GE Healthcare China.

The remaining authors declare that the research was conducted in the absence of any commercial or financial relationships that could be construed as a potential conflict of interest.
