# Supplementary material for: Usefulness of the Texture Signatures Based on Multiparametric MRI in Predicting Growth Hormone Pituitary Adenoma Subtypes
Source: Front Oncol. 2021 Jul 7;11:640375. doi: 10.3389/fonc.2021.640375 (PMC8294058; doi:10.3389/fonc.2021.640375)
Supplement: Supplementary file 1 [file DataSheet_1.pdf]

Supplementary Table 1. List of detailed texture features.

Feature category	Feature list
**Shape (n = 14)**	Volume, surface area, surface area to volume ratio, sphericity, maximum 3D diameter, maximum 2D diameter (column), maximum 2D diameter (row), maximum 2D diameter (slice), major axis, minor axis, least axis, elongation, flatness, mesh volume
**First order statistics (n = 18)**	Energy, Total Energy, Entropy, Minimum, 10th percentile, 90th percentile, Maximum, Mean, Median, Interquartile Range, Range, Mean Absolute Deviation (MAD), Robust Mean Absolute Deviation (rMAD), Root Mean Squared (RMS), Skewness, Kurtosis, Variance, Uniformity
**GLCM (n = 24)**	Autocorrelation, Cluster Prominence, Cluster Shade, Cluster Tendency, Contrast, Correlation, Difference Average, Difference Entropy, Difference Variance, Inverse Difference(ID), Inverse Difference Moment(IDM), Inverse Difference Moment Normalized(IDMN), Inverse Difference Normalized, Informal Measure of Correlation(IMC)1, Informal Measure of Correlation(IMC) 2, Inverse Variance, Joint Average, Joint Energy, Joint Entropy, Maximal Correlation Coefficient(MCC), Maximum Probability, Sum Average, Sum Entropy, Sum of Squares
**GLSZM (n = 16)**	Small Area Emphasis(SAE), Large Area Emphasis(LAE), Gray Level Non-uniformity(GLN), Gray Level Non-uniformity

	Normalized(GLNN), Size-zone Non-uniformity(SZN), Size-zone Non-uniformity Normalized(SZNN), Zone Percentage(ZP), Gray Level Variance(GLV), Zone Variance(ZV), Zone Entropy (ZE), Low Gray Level Zone Emphasis (LGLZE), High Gray Level Zone Emphasis (HGLZE), Small Area Low Gray Level Emphasis (SALGLE), Small Area High Gray Level Emphasis (SAHGLE), Large Area Low Gray Level Emphasis (LALGLE), Large Area High Gray Level Emphasis (LAHGLE)
**GLRLM (n = 16)**	Short Run Emphasis(SRE), Long Run Emphasis (LRE), Gray Level Non-Uniformity(GLN), Gray Level Non-Uniformity Normalized (GLNN), Run Length Non-Uniformity (RLN), Run Length Non-Uniformity Normalized (RLNN), Run Percentage (RP), Gray Level Variance (GLV), Run Variance (RV), Run Entropy (RE), Low Gray Level Run Emphasis (LGLRE), High Gray Level Run Emphasis (HGLRE), Short Run Low Gray Level Emphasis (SRLGLE), Short Run High Gray Level Emphasis (SRHGLE), Long Run Low Gray Level Emphasis (LRLGLE), Long Run High Gray Level Emphasis (LRHGLE)
**NTGDM (n = 5)**	Coarseness, Complexity, Strength, Contrast, Busyness
**GLDM (n = 14)**	Small Dependence Emphasis(SDE),Large Dependence Emphasis (LDE), Gray Level Non-Uniformity (GLN), Dependence Non-Uniformity (DN), Dependence Non-Uniformity Normalized (DNN), Gray Level Variance (GLV), Dependence Variance (DV), Dependence Entropy (DE), Low Gray Level Emphasis (LGLE), High Gray Level Emphasis (HGLE), Small Dependence Low Gray

	Level Emphasis (SDLGLE), Small Dependence High Gray Level Emphasis (SDHGLE), Large Dependence High Gray Level Emphasis (LDHGLE)

GLCM = gray level co-occurrence matrix, GLDM = gray level dependence matrix, GLRLM = gray level run-length matrix, GLSZM = gray level size zone matrix, NTGDM = neighboring gray tone difference matrix
